# The challenge of ovarian tissue culture: 2D versus 3D culture

**DOI:** 10.1186/s13048-021-00892-z

**Published:** 2021-11-01

**Authors:** Ana Sofia Pais, Sandra Reis, Mafalda Laranjo, Francisco Caramelo, Fátima Silva, Maria Filomena Botelho, Teresa Almeida-Santos

**Affiliations:** 1 Reproductive Medicine Unit, Centro Hospitalar e Universitário de Coimbra (CHUC), E.P.E., Coimbra, Portugal; 2 Obstetrics Department, Faculty of Medicine, University of Coimbra, Coimbra, Portugal; 3 University of Coimbra, Coimbra Institute for Clinical and Biomedical Research (iCBR) Area of Environment Genetics and Oncobiology (CIMAGO), Biophysics Institute of Faculty of Medicine, Coimbra, Portugal; 4 University of Coimbra, Center for Innovative Biomedicine and Biotechnology (CIBB), Coimbra, Portugal; 5 Clinical Academic Center of Coimbra (CACC), Coimbra, Portugal; 6 Center for Neuroscience and Cell Biology (CNC), CIBB, Azinhaga de Santa Comba, Celas, University of Coimbra, Coimbra, Portugal; 7 PhD Programme in Experimental Biology and Biomedicine, IIIUC - Institute for Interdisciplinary Research, University of Coimbra, Coimbra, Portugal; 8 Pathology Unit, Centro Hospitalar e Universitário de Coimbra (CHUC), E.P.E., Coimbra, Portugal; 9 University of Coimbra, Faculty of Medicine, Coimbra, Portugal

**Keywords:** Alginate, Ovary, Fertility preservation, Tissue culture techniques

## Abstract

**Background:**

Cryopreservation of ovarian tissue is a powerful technique for preserving female fertility, as it can restore fertility and endocrine function. To increase the longevity of the transplant and decrease the risk of reimplantation of neoplastic cells, several studies have been carried out with culture of ovarian tissue. The aim of this study was to compare a conventional (2D) culture with an alginate matrix three-dimensional (3D) model for ovarian tissue culture.

**Results:**

The ovarian tissue culture within the alginate matrix (3D) was similar to 2D culture, regarding follicular density and cell apoptosis in follicles and stroma. The proliferation rate remained stable in both models for follicles, but for stromal cell proliferation it decreased only in 3D culture (*p* = 0.001). At 24 h of culture, cytotoxicity was lower in the 3D model (*p* = 0.006). As culture time increased, cytotoxicity seemed similar. Degradation of the tissue was suggested by the histological score analysis of tissue morphology after 72 h of culture. Tissue injury was greater (*p* = 0.01) in 3D culture due to higher interstitial oedema (*p* = 0.017) and tissue necrosis (*p* = 0.035).

**Conclusion:**

According to our results, 3D culture of ovarian tissue has no advantage over 2Dculture; it is more time consuming and difficult to perform and has worse reproducibility.

## Background

Over the past decade, cancer incidence has been stable in women, with an overall decline in cancer mortality [1, 2]. This improvement in cancer survival is closely related to the availability of oncological treatments with higher levels of effectiveness, resulting in the focus on issues related to quality of life and long-term survival [3]. Treatments for cancer can be potentially gonadotoxic by directly affecting not only the ovarian follicle pool, but also the ovarian stroma or the blood supply [4]. Thus, premature ovarian failure may arise in patients of reproductive age and, consequently, infertility. Several approaches can be proposed for cancer patients, such as immature/mature oocyte cryopreservation, embryo cryopreservation and ovarian tissue cryopreservation (OTC) [4].

Fertility preservation through OTC is a powerful technique for preserving female reproductive potential; it can preserve thousands of ovarian follicles at once and simultaneously restore endocrine function and fertility, thus allowing spontaneous conception [5, 6] and is the only option for prepubertal girls and women who cannot delay the start of oncological treatments [7]. According to the American Society for Reproductive Medicine (ASRM), this approach is no longer considered experimental [6]. However, there are some concerns about the graft’s survival after transplantation and the potential risk of reimplantation of tumour cells [5, 6]. Therefore, many studies are underway to overcome these limitations and new and experimental techniques have been developed, such as in vitro maturation [8–10].

The ovarian follicles are the morpho-functional unit in the ovary and are composed of an oocyte surrounded by somatic granulosa and theca cells. Basal folliculogenesis is a complex process, involving a dialogue between the oocyte and its closely surrounding cells, through autocrine and paracrine bidirectional signalling [11, 12]. Due to this cross talk, the maintenance of the follicle’s three-dimensional nature during the whole growth process is crucial for the correct acquisition of developmental competence [11]. However, research on in vitro follicle culture has shifted from two dimensional (2D) toward the use of three-dimensional (3D) structures [11]. The use of a matrix maintains the follicle’s 3D architecture and mimics in vivo conditions, with variable access to oxygen and nutrients [11, 13]. This contributes to bridging the gap between conventional cell culture and animal models [14].

Historically, ovarian research is focused on folliculogenesis, but recently the ovarian stroma has become an exciting new field of research, holding critical keys to understanding complex ovarian dynamics [15]. Activation of primordial follicles involves signalling pathways that reach the follicle through microvascularization present in the ovarian cortex [12]. Additionally, stromal tissue also plays an important role for the continued growth of follicles; a vascularized theca differentiates by recruitment of progenitor cells present in the ovarian cortex immediately adjacent to the follicle [12, 16]. Therefore, the culture of fragments of ovarian tissue maintains the integrity and three-dimensional structure of the follicles supporting stromal tissue [16].

By culturing organs in pieces, several cultures from a single organ can be generated, increasing the number of experiments from a single animal [17]. Culture of these tissue types in alginate hydrogels provides mechanical support in order to maintain the three dimensional architecture of the tissue, while the gel itself does not interact with the cells [17].

Alginate offers several advantages over other types of matrices. It is a biocompatible and bioactive natural matrix that floats in standard cell culture medium and provides mechanical support for growing tissues [17, 18].

The aim of this study was to compare a conventional 2D culture with an alginate matrix scaffold for ovarian tissue culture optimization.

## Results

### Tissue morphology and viability

**Fig. 1:**
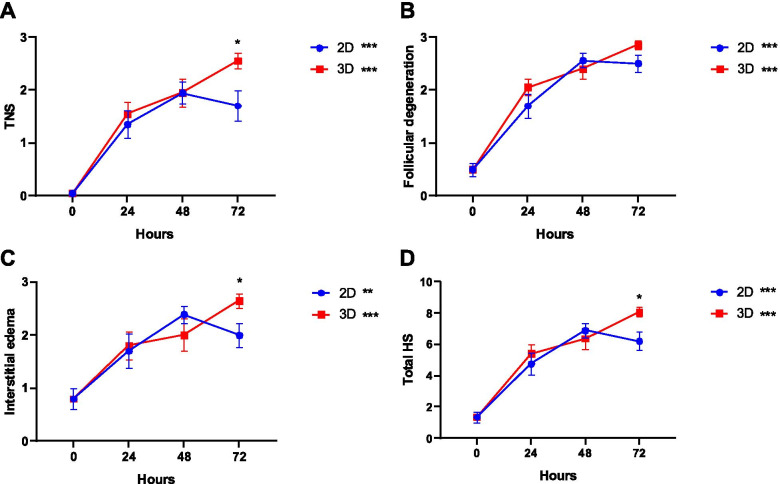
Histopathologic score (HS) results regarding tissue necrosis score (TNS) (**A**), follicular degeneration (**B**), interstitial oedema (**C**) and total HS. A linear regression was performed for 2D and 3D groups and their corresponding slopes show an increase over time. Additionally, a comparison at 72 h was made using the t-test, which shows statistical differences. Data presented as mean ± SEM. *p < 0.05; **p < 0.01; ***p < 0.001

The comparison of histopathologic scores is shown in Fig. 1. During the study period, degeneration of tissue morphology was observed, with a significant increase in the score of all parameters.

According to Fig. 1A, the increase of tissue necrosis score (TNS) is larger in the 3D culture (*p* < 0.001, B = 0.033, *R*^2^_adj_ = 0.663) than for 2D (*p* < 0.001, B = 0.023, *R*^2^_adj_ = 0.381). At 72 h of culture, specimens of the ovary cultured within the alginate matrix (3D) showed higher scores for tissue necrosis (2D = 1.700 ± 0.291 vs. 3D = 2.550 ± 0.138, *p* = 0.017).

The proportion of the degenerated follicles increased over time and was very similar in the two models (2D, *p* < 0.001, B = 0.028, *R*^2^_adj_ = 0.617; 3D, *p* < 0.001, B = 0.031, *R*^2^_adj_ = 0.696; Fig. 1B).

For the score of interstitial oedema (Fig. 1C), a greater increase in the 3D culture was observed (2D, *p* = 0.001, B = 0.018, *R*^2^_adj_ = 0.253; 3D, *p* < 0.001, B = 0.024, *R*^2^_adj_ = 0.428). At 72 h of culture, interstitial oedema was more evident in the 3D group compared to 2D (2D = 2.000 ± 0.224 vs. 3D = 2.650 ± 0.130, *p* = 0.035).

The total histopathologic score (HS), which is the total score of three ovarian injury parameters, increased in both groups but more sharply in 3D culture (2D, *p* < 0.001, B = 0.069, *R*^2^_adj_ = 0.477; 3D, *p* < 0.001, B = 0.088, *R*^2^_adj_ = 0.657; Fig. 1D). Also, after 72 h of culture, the total score was significantly higher in 3D culture than 2D culture (2D = 6.200 ± 0.569 vs. 3D = 8.050 ± 0.302, *p* = 0.01).

**Fig. 2:**
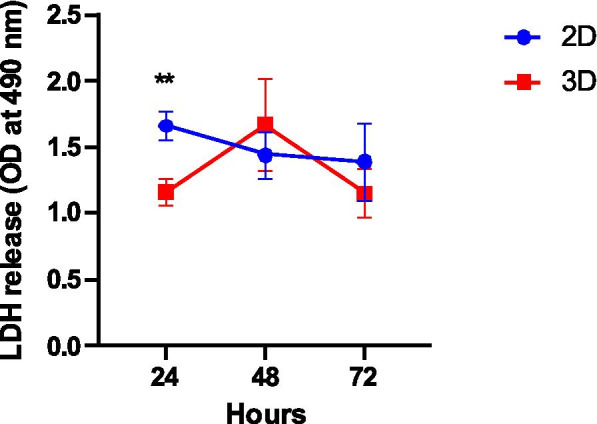
Cytotoxicity analysed through the release of lactate dehydrogenase (LDH) into culture supernatants. Data presented as mean of optical density (OD) ± SEM. A comparison at 24 h was made using the t-test, which shows statistical differences. **p < 0.01

During the study period, there were no changes in the amount of lactate dehydrogenase (LDH) released into the culture medium (Fig. 2). The amount of LDH released was lower in culture with the alginate matrix than in conventional culture (1.16 ± 0.10 vs. 1.66 ± 0.11, *p* = 0.006), after 24 h. However, no differences were found at 48 and 72 h of culture.

### Follicular analysis

**Fig. 3:**
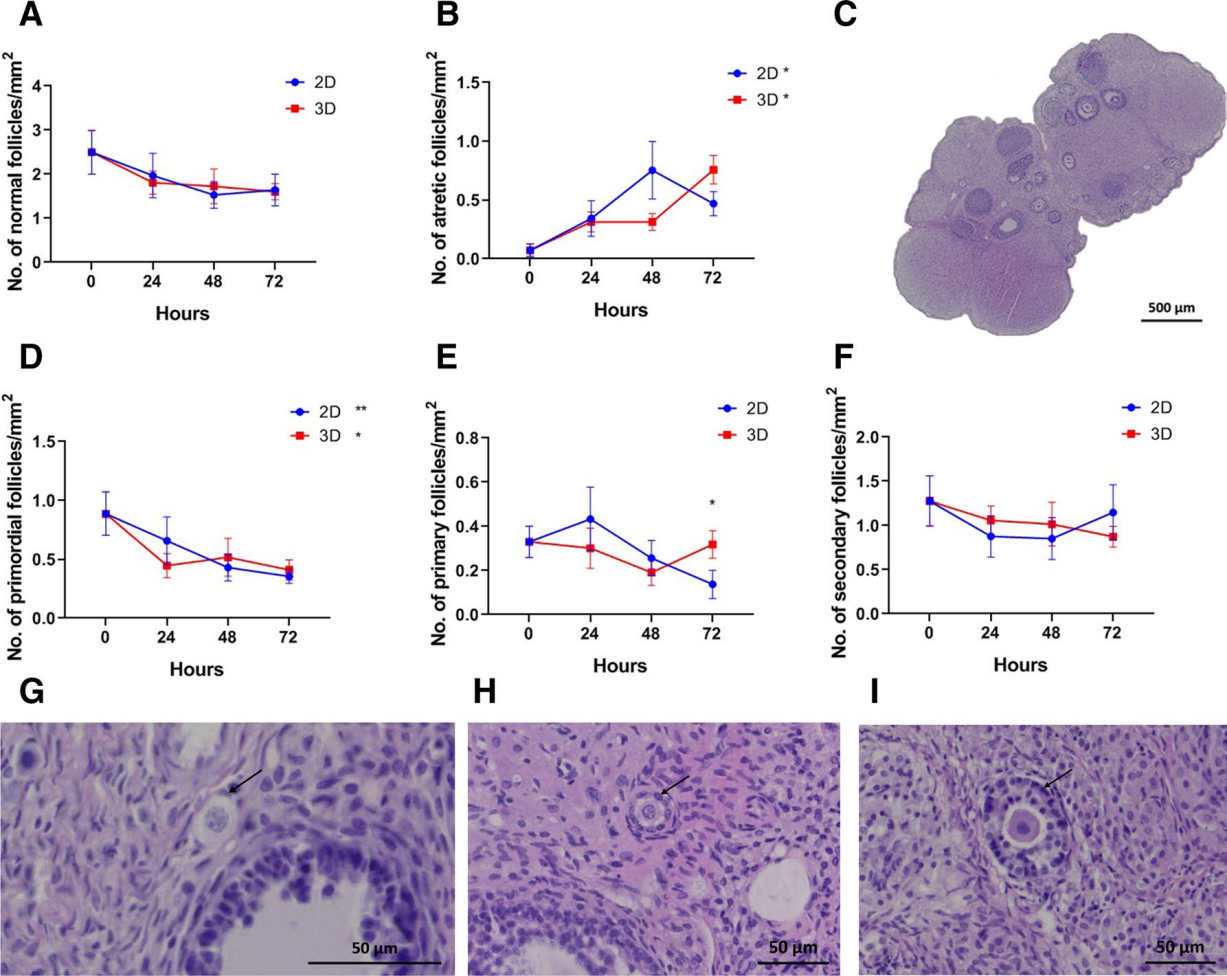
Follicular analysis. Representative images of ovarian tissue (**C** scale bar: 500 μm) and morphological normal primordial (**G**), primary (**H**) and secondary (**I**) follicles (arrow, scale bar: 50 μm). Density of follicles classified as normal (**A**) and degenerated (**B**) according to oocyte and granulosa cell morphology. Density of primordial (**D**), primary (**E**) and secondary (**F**) follicles. A linear regression was performed for 2D and 3D groups and their corresponding slopes show an increase over time in atretic follicles and a decrease in primordial follicles. Additionally, a comparison between 2D and 3D at 48 and 72 h was made using the t-test, which shows statistical differences for the density of primary follicles at 72 h. Original magnification: A × 100; B × 400; H and I × 200. Data presented as mean ± SEM. *p < 0.05; **p < 0.01; ***p < 0.001

From the histological evaluation, we observed that density of morphologically normal follicles was maintained during the 72 h of culture (Fig. 3A). However, the density of atretic follicles increased significantly in both groups (2D, *p* = 0.027, B = 0.007, *R*^2^_adj_ = 0.102; 3D, *p* < 0.001, B = 0.009, *R*^2^_adj_ = 0.412; Fig. 3B).

Regarding follicular classification, there was a significant decrease of primordial follicles in both types of culture (2D, *p* = 0.009, B = − 0.008, *R*^2^_adj_ = 0.169; 3D, *p* = 0.036, B = − 0.006, *R*^2^_adj_ = 0.11; Fig. 3D and G). The density of primary and secondary follicles remained the same during the 72 h of culture (Fig. 3E, F, H and I, respectively). At 72 h of culture, the density of primary follicles was significantly higher in the samples cultured with the alginate matrix (2D = 0.138 ± 0.064 vs. 3D = 0.318 ± 0.063 follicles/mm^2^, *p* = 0.035; Fig. 3E).

### Proliferation and apoptosis in ovarian tissue

**Fig. 4:**
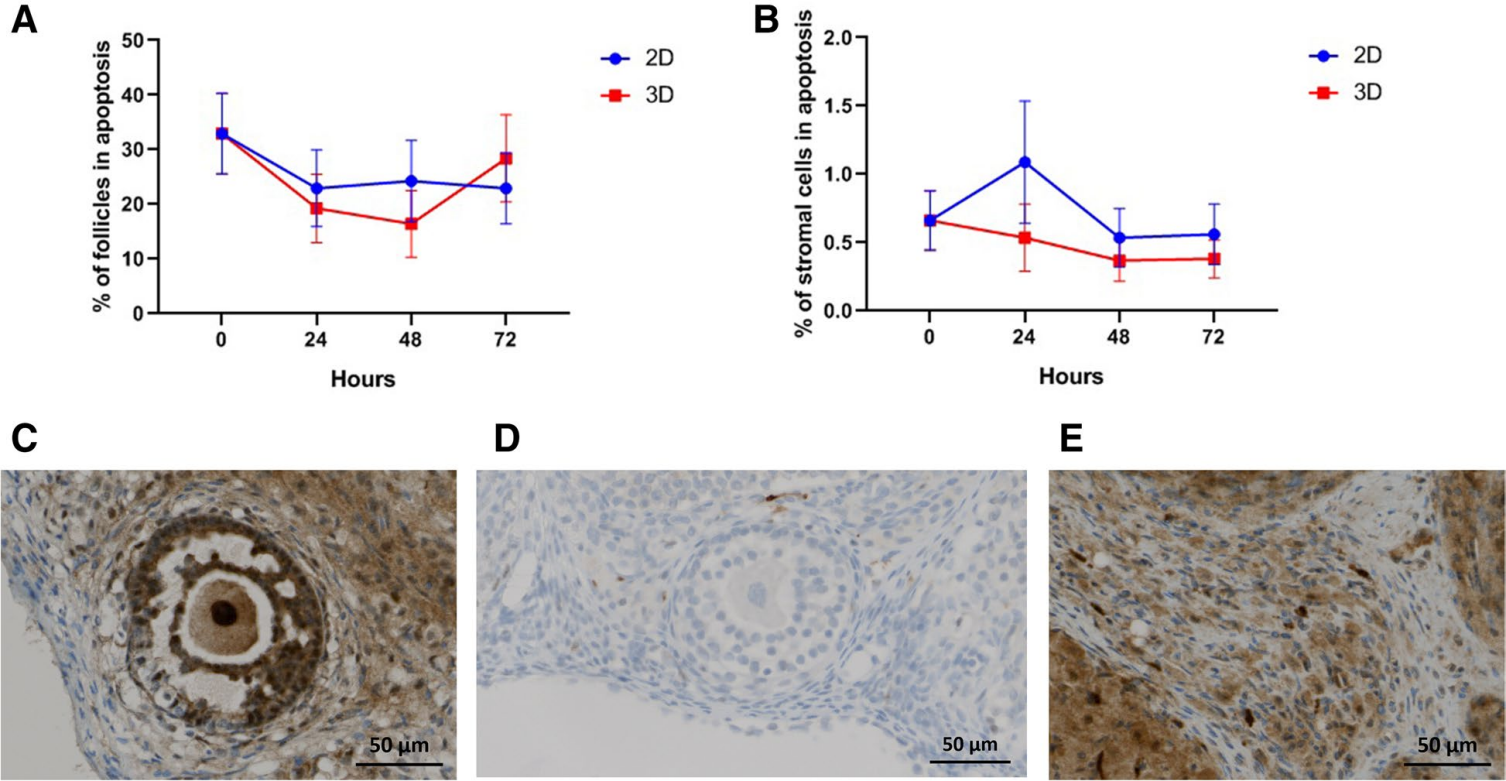
Tissue apoptosis evaluated through caspase-3 staining in follicles (**A**) and stromal cells (**B**). Representative images of AC-3 staining in a secondary follicle (**C**) and stromal cells (**E**). A negative secondary follicle is represented in **D**. Original magnification × 200 and scale bar 50 μm (**C**, **D** and **E**). Data presented as mean ± SEM

The levels of apoptosis assessed by the percentage of caspase-3 positive follicles and stromal cells remained stable during the culture period; no significant differences were found (Fig. 4).

**Fig. 5:**
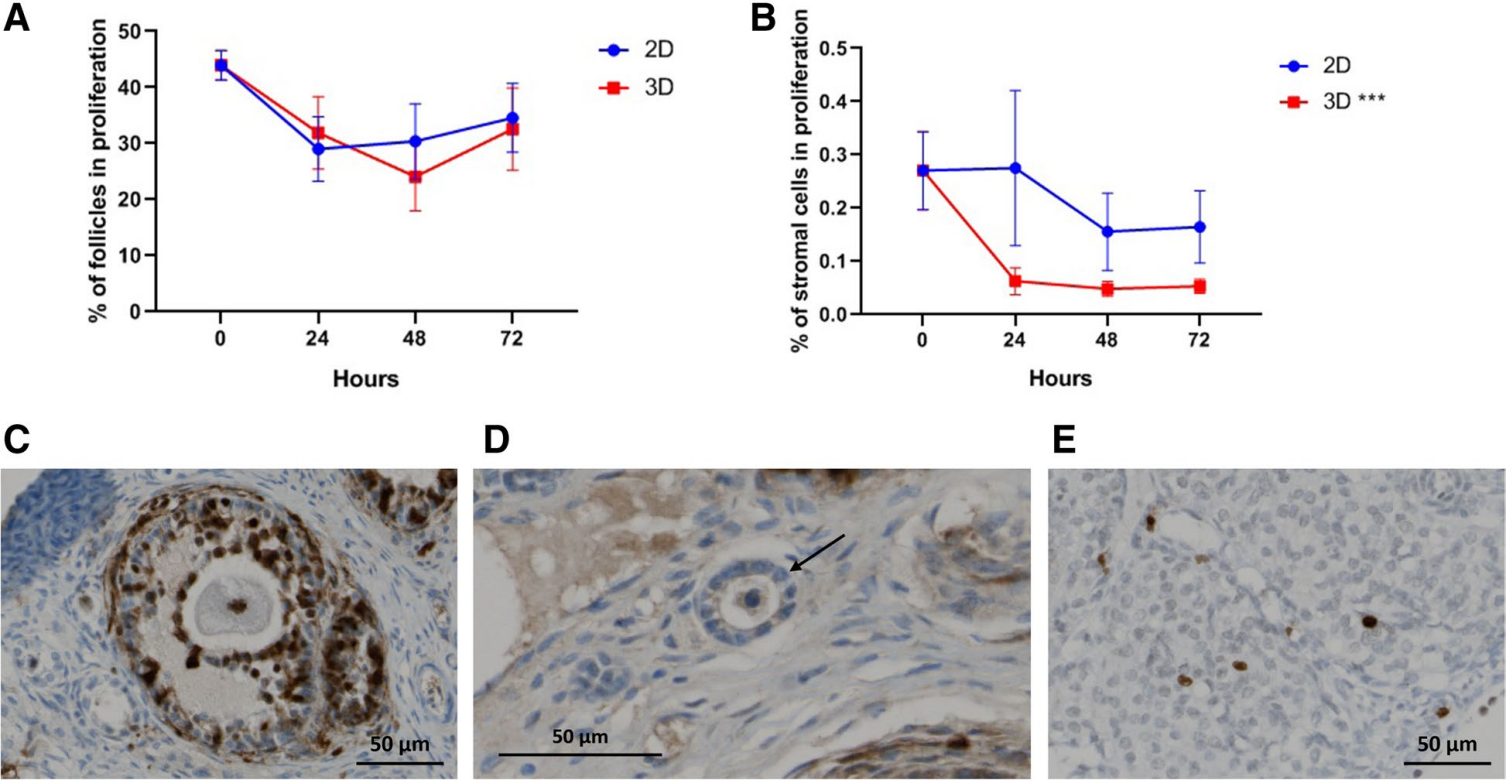
Tissue proliferation evaluated through Ki67 staining in follicles (**A**) and stromal cells (**B**). Representative images of Ki67 staining in a secondary follicle (**C**) and stromal cells (**E**). A negative primary follicle (arrow) is represented in **D**. A linear regression was performed for 2D and 3D groups and their corresponding slopes show a decrease over time in stromal cell proliferation for 3D group. Original magnification × 200 (**C**, **E**) and × 400 (**D**), scale bar 50 μm. Data presented as mean ± SEM. ***p < 0.001

The percentage of proliferative follicles, assessed with Ki67 staining, remained stable during the culture period in both groups (Fig. 5A, C and D). However, the evaluation of proliferation in stromal cells revealed a significant decrease in the 3D culture (*p* = 0.001, B = − 0.003, *R*^2^_adj_ = 0.220; Fig. 5B and E).

## Discussion

Cryopreservation of ovarian tissue has been considered a reliable possibility for preservation of fertility in young women at risk of iatrogenic premature ovarian failure due to oncological treatments [19]. This is the only option for prepubertal girls and women who cannot delay the beginning of chemotherapy or cannot undergo an ovarian stimulation protocol for oocyte or embryo cryopreservation [19, 20].

The cryopreservation of ovarian tissue began in the 1950s; in the following years, several improvements were accomplished in the post-thaw viability of rat and mouse ovarian tissue. But only in the 1990s, after the successful delivery of a live birth from a frozen-thawed mouse primordial follicle [21], was this technique envisaged as an option for preservation of fertility [22].

The possibility of achieving complete oocyte development in vitro*,* in primates and most domestic species, has boosted research in this field. Different options were evaluated in order to overcome technical difficulties and an in vitro system was developed for culturing small pieces of ovarian cortex [20, 23]. The cortical fragments are the main reservoir of dormant primordial follicles in the postnatal ovary, which determines the woman’s fertile lifespan [20, 24].

Many culture approaches have been developed for different animal models [25]; however, the optimal model has not been achieved. Further efforts are needed in order to establish a reliable model for tissue culture [23, 26]. Higuchi et al. described the in situ ovarian tissue culture as an innovative and promising methodology to grow follicles in vitro due to its easy and safe handling, and more importantly the possibility of reconstituting the ovarian microenvironment or manipulating some conditions to induce follicle growth [27, 28].

Our work aimed to evaluate some drawbacks concerning the culture of ovarian tissue in a 3D model. In this model, OT was cultured in an alginate matrix, which has been widely used for the in vitro culture of isolated follicles in mice [29, 30] and rats [31]. The biochemical properties of this matrix make it a good candidate for 3D models. It has gained recognition for: its physicochemical and biological properties, such as its ability to make hydrogels under physiological conditions, providing little disruption to the tissue when encapsulated; the fact that the dissolution of gels for cell/tissue retrieval is easy to control; and its transparency for microscopic evaluation [17]. The physical features of the alginate hydrogel pore network allow the diffusion of nutrients and waste materials, namely the bidirectional diffusion of hormones and other proteins that are essential for tissue survival and also follicular development [32, 33]. Also, it is not of animal origin, which makes it biocompatible [33].

The histopathological evaluation of ovarian tissue (Fig. 1) elicited a global deterioration of tissue viability, and this decrease is time dependent. Tissue deterioration was observed in both models, with no differences between 2D and 3D within the first 48 h. This is in accordance with other previously published works that report no difference in cell viability in the first days of culture in the 2D and 3D models [34]. However, after 72 h there is a significant decrease in tissue viability in the 3D model. A decrease in tissue viability when the culture time is prolonged was previously described and a possible explanation for this is that the organ structure might retain the cellular waste produced and possibly induce a shortfall in the supply of oxygen and nutrients [35]. In the 3D model, the architecture is complex, with the tissue presenting three layers: (i) an outer layer with a high proliferation rate, (ii) a middle layer with senescent cells and (iii) a hypoxic core; this organization affects the supply of oxygen and nutrients, retaining the cellular waste produced [35, 36].

To measure cell death, LDH assay was performed in culture medium; this assay is based on the ability of LDH to convert pyruvate to lactate while consuming NADH [37]. In our data, we demonstrate that it is possible to detect and quantify LDH in the medium in both models, as previously described [37]. In the first 24 h, the 3D model presents a low cytotoxicity when compared with conventional culture. At 48 h and 72 h hours of culture, no difference was observed in the amount of LDH released in culture medium for both models. This effect may occur due to LDH retention in the alginate matrix. Although alginate matrix has higher porosity, allowing the diffusion of small substrates (molecular weight (MW) < 20 kDa) at the same speed as water [38], some proteins with higher MW, such as albumin (MW = 69 kDa), have a lower diffusion. Our results suggest that LDH (140 kDa) [39] may be retained longer in the spheroid structure. The diffusion resistance of alginate matrix was described for the first time, in 2006, in HepG2 cells encapsulated in alginate beads [37].

In order to establish a more detailed histopathological evaluation, we developed a score, based on the evaluation of three histologic parameters: TNS, interstitial oedema and follicular cell degeneration [40–42]. TNS and interstitial oedema are closely related parameters. Necrosis is characterized as an unprogrammed cell death process, which begins with cell swelling and results in cell membrane rupture and release of cell cytoplasmic content into the extracellular space [43], creating an extracellular movement of fluids. Our data show an increase in TNS and in interstitial oedema over experimental time in both models. However, after 72 h the increase in both parameters was greater in 3D culture. In the interior of the 3D structure, the bioavailability of oxygen and nutrients may be limited, affecting cell survival over time and conditioning higher level of necrosis and release of intracellular content when compared to more superficial cells [35]. In our characterization of tissue degeneration in the in vitro culture models, we detected an abundant level of follicular degeneration (Fig. 1B), and no differences were observed in the 2D model versus the 3D. A more detailed analysis reveals an increase in follicular atresia (Fig. 3B) accompanied by a decrease in primordial follicles (Fig. 3D). Our results are in line with previous work, which showed a time-related increase of follicular atresia in culture [44].

The biocompatibility of alginate matrix was intensively investigated using in vitro and in vivo models, and it was described as not inducing an immune response [45], first because of the absence of cell receptors to recognize alginate and second due to the high-purity commercialized alginate. This compound is extracted from brown algae, and may contain various impurities such as heavy metals, endotoxins, proteins, and polyphenolic compounds [46]; however, multi-step purification ensures a high level of purity in the alginate. Despite the safety of this compound, its widespread use has been challenged by some drawbacks concerning the molecular size, charges and viscosity of alginate, different culture models, different implantation sites and different animal models employed [45].

A different proliferation rate is observed when 2D and 3D models are compared. As shown in Fig. 5B, the proliferation rate of stromal cells is decreased in the 3D model. The difference in the proliferation rates between 2D and 3D models was previously described; cell lines cultured in the 3D system showed a reduced proliferation when compared with the 2D model, which proliferate at an unnaturally rapid rate [14]; these effects are dependent on the 3D model employed, and more specifically are matrix dependent, and also dependent on specific cells properties [14, 36]. In the field of fertility preservation, this effect was also reported in follicular in vitro culture; some authors have described that the physical properties of alginate matrices can limit the growth and development of earlier secondary follicles [17, 28, 30]. However, this topic is very controversial, and it is continuously under review. Different reasons have been suggested to explain these findings. In the context of our work, the rigidity of alginate matrix may be a possible explanation for the decreased proliferation rate [27]. Softer matrices with lower alginate concentrations [30] or the combination of different elements in the matrix to allow more space and the diffusion of macromolecules responsible for the tissue survival and growth were proposed [28].

Additionally, we evaluated cellular death using a caspase-3 antibody as a marker for programmed cell death. Caspase-3 is a well characterized protease which plays an effective role in apoptosis [47, 48]. The caspase-3 expression was already studied in granulosa [49] and theca cells [50] and also in oocytes [44]. In our study, apoptosis was evaluated in stromal cells and follicles, with the level of apoptosis in stromal cells and follicles being stable during the experimental time with no difference between conditions (2D vs. 3D model) (Fig. 4). However, it is important to keep in mind that apoptosis is a central process in ovarian function and development and occurs from foetal life on; it mainly affects the oocyte, until adult life when it affects the granulosa cells in growing follicles [47, 51].

In this study, we performed a well-structured histopathological analysis and the major strengths of our study are the sample size and the duplicate and blind analysis of results. However, some points could be addressed in future research to step up the 3D model, namely the study of the tissue-matrix interactions and culture medium supplementation to decrease follicular atresia.

## Conclusions

In conclusion, the ovarian tissue culture within an alginate matrix was similar to 2D culture, regarding follicular density, follicular cell proliferation and cell apoptosis in follicles and stroma. In 3D culture, greater levels of tissue injury and oedema and lower stromal cell proliferation were seen. Therefore, there is no clear advantage in the 3D culture of ovarian tissue, as it is more time-consuming, difficult to perform and less reproductible.

## Methods

### Ethical statement

The present study was approved by the Ethics Committee for Animal Experimentation (ORBEA Authorization number 11060495/23-11-2016) of the Faculty of Medicine of the University of Coimbra and performed according to European Guidelines and Portuguese Law. The Guide for the Care and Use of Laboratory Animals of the National Institutes of Health was followed to take care of the animals and the ARRIVE guidelines to perform and report the experimental protocols.

### Study design

**Fig. 6:**
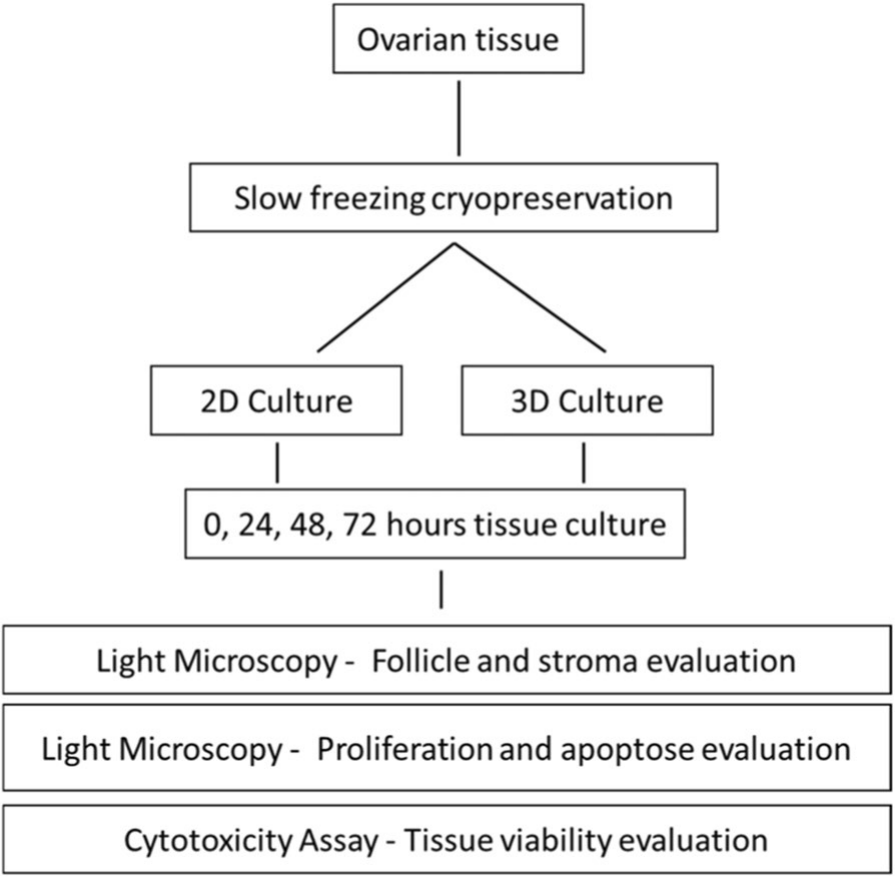
Schematic representation of the study. The figure shows the experimental design of the comparative study of in vitro culture system for ovarian tissue – 2D versus 3D

The experimental scheme is shown in Fig. 6. Briefly, ovarian tissue (OT) preparation, cryopreservation, 2D system or encapsulated model procedure and timescale for the experiment are shown.

### Experimental animals

The animal facility of the Faculty of Medicine of the University of Coimbra provided 12 female Rowett nude rats (RNU, homozygous) aged 8-10 weeks and with an average weight of 200 g. Housing, under a 12-h light/dark cycle, was in individually ventilated cages, with access to standard diet and filtered water ad libitum. Animals were distributed in a randomized manner into the study groups described below.

### Ovaries collection

Surgical procedures were performed under inhalation anaesthesia with sevoflurane (5%) and with subcutaneous analgesia with carprofen (5 mg/kg, making a total of 0.2 mL per animal).

For bilateral ovariectomy, animals were placed in a supine position and the abdominal wall was shaved, cleaned and sterilized with povidone-iodine solution. A longitudinal median laparotomy was performed with a 2–3 cm incision in the lower area of the abdomen. The ovaries were identified and removed with the ligation of the vascular pedicle [52, 53]. After ensuring adequate haemostasis, the abdominal cavity was closed in layers.

After ovariectomy, the ovaries were placed in cold Dulbecco’s phosphatase-buffered solution (DPBS) (Biological Industries, Sartorius, Gottingen, Germany) supplemented with 10% foetal bovine serum (FBS) (Biological Industries, Sartorius, Gottingen, Germany) and each ovary was cut in 2 hemi-ovaries (3 × 2 × 1 mm). The fragments were maintained for 30 min in cold DPBS + 10% FBS before cryopreservation.

### Cryopreservation and thawing protocols

The cryopreservation protocol consisted of a slow freezing and a rapid thawing method, adapted from Rosendahl M et al. [54]. The fragments were placed in plastic cryovials (Nunc, Thermo Fisher) containing 1.5 mL of freezing media consisting of 1.5 M ethylene glycol (Sigma-Aldrich, St. Louis, Missouri, USA), 0.1% sucrose (Merck, Darmstadt, Germany) and 10 mg/mL human albumin serum (HAS) (Grifols, Barcelona, Spain), and maintained in an ice bath. The cryovials were transferred to a rolling system for 30 min at 4 °C to allow the cryoprotectant to enter the tissue. Then, cooling in a programmable freezer (Planner cryo 10 Series 2 Freezer) followed the subsequent protocol. The starting temperature was 0 °C, and it was slowly reduced to − 9 °C at a rate of − 2 °C/min. After a 5-min holding time at − 9 °C, manual seeding was performed, after then the cryovials were cooled to − 40 °C, at a rate of − 0.3 °C/min, and the final step, a rapid decrease to − 140 °C (− 10 °C/min). When the programme was completed, cryovials were transferred into liquid nitrogen tank and stored for 1 week until thawing.

On the day of the experiment, ovarian fragments were thawed. The vials were air-warmed for 30 s and then immersed in a 37 °C water bath for 5 min. The freezing media was removed at room temperature by stepwise dilution of freezing media in sequential thawing media stabilized at room temperature. Three culture dishes were filled with thawing medium I (0.75 M ethylene glycol + 0.25 M sucrose in PBS + 10 mg/mL HAS), medium II (0.25 M Sucrose in PBS+ 10 mg/mL HAS), and medium III (PBS + 10 mg/mL HAS). Ovarian fragments were transferred into thawing medium I using sterile forceps and stirred for 10 min at room temperature. The same procedure was performed for thawing media II and III. Thawed tissue was transferred to PBS before culture.

### Ovary culture

Hemi-ovaries (3 × 2 × 1 mm) were cultured in the growth media with and without encapsulation in an alginate matrix scaffold. The conventional culture was defined as 2D and the use of the scaffold as 3D. A 1.5% (w/v) solution of sodium alginate (Sigma-Aldrich, St. Louis, Missouri, USA) was prepared by mixing into sterile DPBS and heating to 37 °C. To encapsulate the ovarian fragments, the agarose (Invitrogen, California, USA) ring protocol was used, adapted from Henry, 2015 [55]. Agarose rings were filled with a layer of alginate matrix, fragments were placed in the ring and they were covered with matrix. Cross-linking solution (50 mM CaCl_2_ + 140 mM NaCl) was added, allowing the solution to solidify into a gel around the ovarian organoid.

The gel-organoid was then placed in the growth media to be cultured for 24 h, 48 h and 72 h. The growth media consisted of α-MEM (22561-021, ThermoFisher, Waltham, Massachusetts, USA), 10% FBS and 1/1000 penicillin/streptomycin (15140-122, Gibco, ThermoFisher, Waltham, Massachusetts, USA).

### Tissue morphology and viability

The ovaries were fixed in 4% formaldehyde (Panreac Quimica Sau, Barcelona, Spain), embedded in paraffin, and sectioned serially at 5 μm. Three sections per transplant were stained with haematoxylin and eosin (HE) for morphological analysis. The images were acquired on the Axioscan Z1 (Carl Zeiss), with a Plan-Apochromat 10x/0.8 lens, and photographed with the Zen 2 program blue edition (Carl Zeiss Microscopy GmbH, 2011). Histological analysis was performed blindly by a researcher, at two different points in time, using the Image J software.

**Fig. 7:**
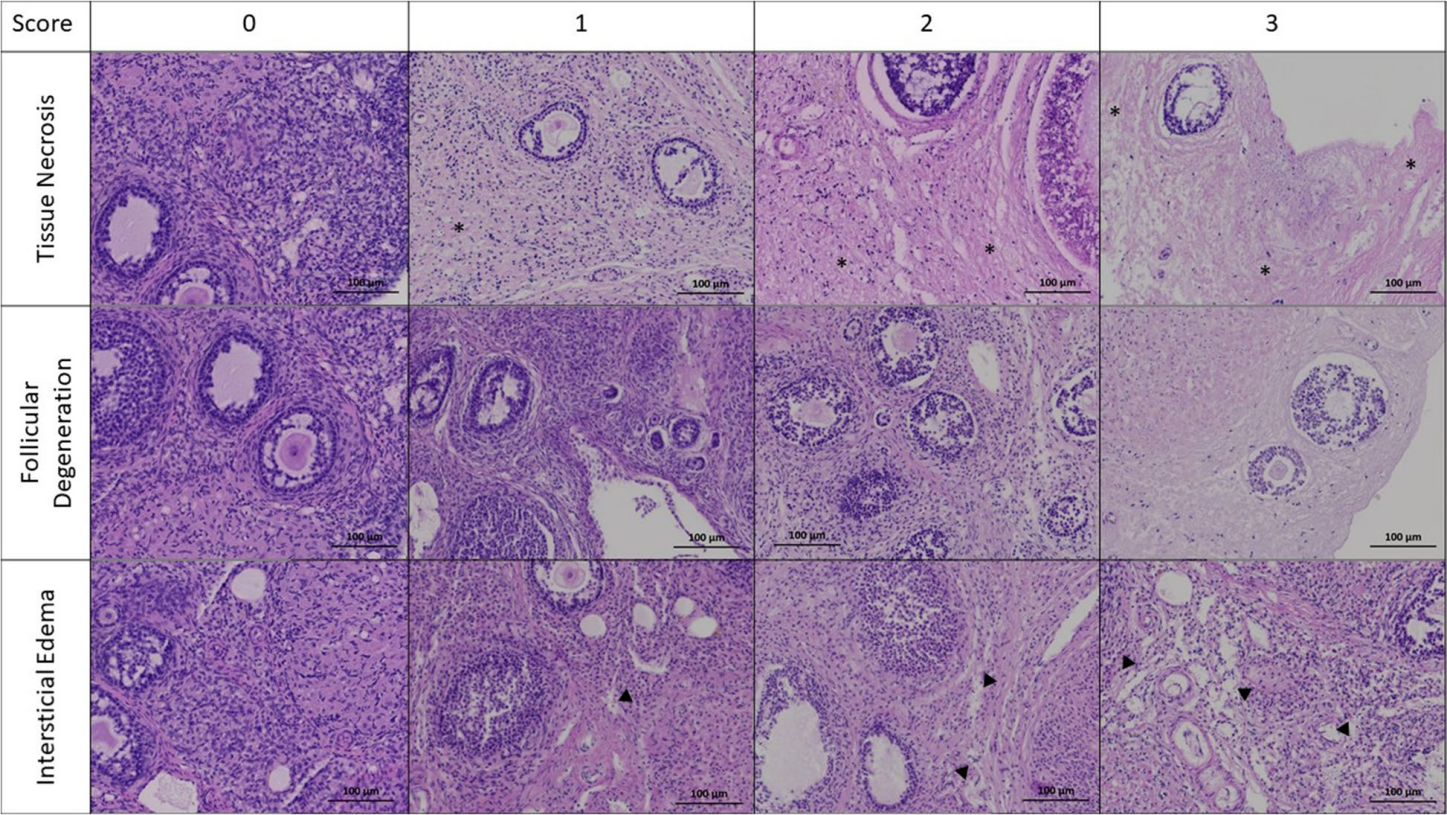
Histopathologic score (HS) represented in haematoxylin and eosin-stained ovarian sections. The images show areas of tissue necrosis (*), follicular degeneration and interstitial oedema (▼). A scoring system where none = 0, mild = +, moderate = ++ and severe = +++ was applied. Original magnification × 100 and scale bar 100 μm

The criteria for ovarian tissue viability was adapted from criteria previously described for ovarian tissue injury after in vivo experiments of ischemia/reperfusion [40–42, 56]. As shown in Fig. 7, histopathological examination of the tissue damage was performed in terms of three visual parameters: interstitial oedema, follicular cell degeneration and percentage of tissue in necrosis (TNS).

The follicles were histologically classified as degenerated when they included cells with pyknotic nucleus, shrunken ooplasm and disorganized granulosa cells. Follicular degeneration score was calculated as a proportion of the degenerated follicles to the total number of follicles [56]. Oedema is analysed by the subjective quantity of intercellular liquid. The extent of overall necrosis within each ovary was quantified by a visual assessment of the percentage of area in necrosis (TNS) for each specimen [40]. Necrotic cells typically present cytoplasmic hypereosinophilia, cellular swelling, blebbing, and loss of microvilli [57]. Each parameter was scored using a scale ranging from 0 to 3 (0, none; 1, mild or < 33%; 2, moderate or 33–66%; 3, severe or > 66%) [41]. Total scores were calculated according to these parameters [41]. Ovary sections were analyzed double blind.

### Follicular analysis

Follicles were quantified manually and, to avoid double counting, only follicles with a visible nucleus were taken into account. Only morphologically normal follicles were taken into consideration for quantitative analysis. Follicles were then classified according to their maturity as primordial (constituted by a single layer of flattened granulosa cells), primary (they present a single layer of cuboid granulosa cells) and secondary (composed by two or more layers of granulosa cells around the oocyte) [58]. The pre-antral and antral follicles were grouped with the secondary follicles. Follicle atresia was assessed with morphologic criteria, such as irregular shape, granulosa cell pyknosis, cytoplasmic contraction, presence of vacuoles and ooplasm eosinophilia [58]. Follicular densities were calculated as the number of follicles per area (number/mm^2^), after measuring the tissue by manual surrounding of the surface (Fig. 3C) [59].

### Proliferation and apoptosis evaluation

Immunohistochemistry assay was performed with the cell proliferation biomarker rabbit polyclonal antibody Ki-67 (diluted 1:300; PA5-19462, Thermofisher, Waltham, Massachusetts, USA) and cell apoptosis rabbit polyclonal antibody caspase-3 (diluted 1:100; AHP2286, Bio-Rad Laboratories, Hercules, California, USA). Antigen retrieval was performed with CC1, pH 8 EDTA/Tris-based buffer (Ventana Medical Systems, Tucson, Arizona, USA), followed by primary antibody incubation according to the manufacturer’s procedures and the dilutions previously mentioned. Detection of immunostaining was performed with OptiView DAB IHC Detection Kit (Ventana Medical Systems, Tucson, Arizona, USA) in accordance with the manufacturer’s recommendations, by the detection of DAB chromogenic substrate precipitated. All the immunostained sections were then nuclear counterstained with haematoxylin, dehydrated in a graded series of ethanol, cleared in xylene and mounted using a synthetic mounting medium.

After the immunohistochemistry protocol, in which the incubation with the cell proliferation indicator (Ki-67) and cell apoptosis (caspase-3), the images were acquired on the Axioscan Z1 (Carl Zeiss), with a Plan-Apochromat 10x/0.8 lens and photographed with the aid of the Zen 2 blue edition program (Carl Zeiss Microscopy GmbH, 2011). The results analysis was double and blinded.

Follicles were classified as positive or negative for Ki67 and caspase-3 (Figs. 4C, D and 5C, D). For this, positivity was considered when staining was observed in the oocyte and/or in at least one granulosa cell [60, 61]. Regarding the stroma, the quantification of the area marked with each antibody was performed using the Image J program (Fiji version, 1.8.0, USA). Five zones of each sample were randomly selected through the application of a rectangular grid and the results subsequently presented as an average (Figs. 4E and 5E).

### Cytotoxicity evaluation

To evaluate the tissue viability, the lactate dehydrogenase (LDH) released in the culture medium from damaged cells was measured using CytoTox96® non-radioactive cytotoxicity assay (Promega G1780, Madison, Wisconsin, USA) [62–64]. The assay was performed according to the manufacturer’s protocol. Briefly, after 24 h, 48 h and 72 h of culture, the supernatant of each condition was collected and stored at − 20 °C. On the day of LDH assay, the CytoTox96® reagent was prepared mixing the buffer assay with the substrate mix. To test the effects of 2D vs. 3D culture, 50 μL of culture medium was transferred to a 96 multi-well, flat, clear-bottom plate and mixed with 50 μL CytoTox96® reagent and incubated for 30 min, protected from light. After that, 50 μL of stop solution was added to each well, and the optical density at 490 nm was measured using an EnSpire microplate reader. The absorbance values from the conditioned media supernatant were normalized to mean absorbance values calculated from control samples.

### Statistical analyses

The number of ovaries to be used was estimated using the G*Power software version 3.1.9.4 (Kiel, Germany). A comparison of results between the seven described groups was considered, with a type I error of 0.05 and a statistical power of 0.90, for an effect size of 0.60. In this way, a total sample size of 66 fragments (9.4 per group) was estimated, with an effective power of 0.901. Therefore, 10 hemi-ovaries were used per group.

Statistical analyses were performed using the SPSS version 22.0 (IBM, Armonk, New York, USA). To compare the 2D and 3D groups, simple linear regression over time was performed using a bootstrapping strategy (1000 samples). The regression coefficient (B), which represents the slope, was reported. A positive B-value means an increase over time, while a negative one means a decrease. For example: *p* < 0.001, B = 0.033, *R*^2^_adj_ = 0.663, means that per hour there is a significant increase of 0.033 (B) in the analysed variable and the linear model explains 66% (R^2^_adj_) of the variation over time. Normal distribution was then evaluated to compare the two study groups (2D vs. 3D), which visually showed a reasonable difference. Normally distributed variables were compared by means of the Student t-test and non-normally by means of a Mann-Whitney test. A *p*-value of less than 0.05 was considered statistically significant. The results are expressed as mean ± standard error of the mean (SEM).

## Data Availability

Not applicable.

## Abbreviations

2D: Two-dimensional
3D: Three-dimensional
ASRM: American Society for Reproductive Medicine
DPBS: Dulbecco’s phosphatase-buffered solution
FBS: Foetal bovine serum
HAS: Human albumin serum
HE: Haematoxylin and eosin
HS: Histopathologic score
LDH: Lactate dehydrogenase
OT: Ovarian tissue
OTC: Ovarian tissue cryopreservation
SEM: Standard error of the mean
TNS: Tissue necrosis score
